# Gas sensor for 4-ethylguaiacol detection based on tyrosinase enzymatic activity in a deep eutectic solvent

**DOI:** 10.1007/s00604-025-07218-6

**Published:** 2025-05-20

**Authors:** Paula Portugal-Gómez, Rossella Svigelj, Fabiola Zanette, Rosanna Toniolo, Olga Domínguez-Renedo, M. Asunción Alonso-Lomillo

**Affiliations:** 1 Analytical Chemistry Department, Faculty of Sciences, University of Burgos, Pza. Misael Bañuelos S/N, 09001 Burgos, Spain; 2 Department of Agrifood, Environmental and Animal Sciences, University of Udine, Via Cotonificio 108, 33100 Udine, Italy

**Keywords:** Phenols, Screen-printed carbon electrodes, Nanomaterials, Gas sensor, Deep eutectic solvents, Wine analysis

## Abstract

The use of environmentally friendly, cost-effective and biodegradable deep eutectic solvents (DESs) as nonaqueous solvents and electrolytes offers a promising avenue for enhancing enzymatic sensors and extending their applicability to the gas phase. In this study, an assembly is presented that includes a paper crown modified with tyrosinase enzyme, soaked in a DES and positioned on a disposable screen-printed carbon electrode. The paper crown contacts the outer edge of the carbon disk working electrode, as well as the peripheral counter and reference electrodes. This assembly yields a portable and disposable electrochemical platform, effortlessly immobilising DESs onto a porous and economical supporting material like paper. Moreover, the entire configuration resulted in a sensitive, rapidly responsive, membrane-free gas sensor whose response time depended exclusively on the enzymatic reaction. The electroanalytical capabilities of this setup were evaluated through voltammetric and amperometric determinations of phenols in synthetic and real wine samples. The proposed gas enzymatic sensor demonstrated excellent analytical performance for detecting phenolic compounds, offering fast measurement times, simple operation, high sensitivity, wide linear range and good repeatability.

**Graphical Abstract:**

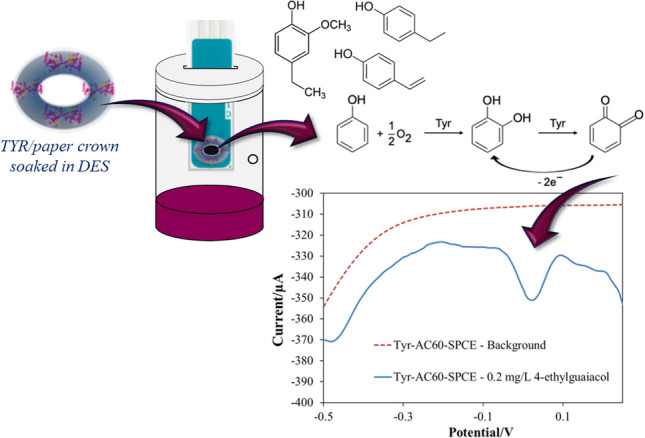

## Introduction

Volatile phenols, such as 4-ethylphenol, 4-ethylguaiacol and 4-vinylphenol, can degrade the quality of wine due to unpleasant odours. In fact, organoleptic properties, which include taste, smell and colour, are key indicators of quality, which is crucial so that wine can stand out among the high production available nowadays and be chosen by consumers. The presence of these volatile compounds in wines can lead to product returns and loss of confidence in the brand, causing significant economic losses in the wine industry, which has become extremely important at the social, cultural and economic levels of many regions. Thus, monitoring phenol levels helps winemakers assess wine stability and quality, prompting interventions such as filtration or sulphur dioxide addition to manage microbial contamination and prevent spoilage [1]. Compared to chromatographic analytical methods, electroanalytical sensors are highly effective tools for diverse applications, not only because of their inherent selectivity and sensitivity, but also because they facilitate in situ measurements within the same environment as the analyte with cost-effective instrumentation [2–4]. Carbon paste, glassy carbon and screen-printed electrodes modified by different compounds, such as metallic nanoparticles (NPs), metal oxide NPs, fullerene (C60), polymers and molecularly imprinted polymers, have been reported for the analysis of these compounds in wine [5–8]. For the quantification of 4-ethylguaiacol in wines, an extraction pretreatment with ether was necessary to successfully quantify this compound owing to the complexity of these samples [8].

Conducting electrochemical measurements of these volatile phenols in the gas phase would avoid spoilage of wine samples, as it does not require any contact with the electrode. Furthermore, faster response times, owing to the faster diffusion rates of gases, and enhanced selectivity, because non-volatile compounds will not reach the working electrode, are provided in this way [9–11]. However, problems arise when these devices operate in the gas phase because they are usually in a dry environment, where the aqueous electrolyte tends to evaporate, which can eventually affect the response, stability and lifetime of the sensor [12]. Significant advantages can be harnessed by employing non-aqueous electrolytes in electroanalytical applications such as room-temperature ionic liquids or deep eutectic solvents (DESs). Although both electrolytes have shown similar performance in gas sensors [13], DESs have been defined as a cheaper and greener alternative [14–17]. DESs result from the mixing of the hydrogen bond acceptor (HBA) and hydrogen bond donor (HBD) components in stoichiometric ratios. Notably, they exhibit negligible volatility, good conductivity, high thermal stability and customisable chemical-physical properties (polarity, viscosity, conductivity, electrostability range, melting point, density) through the careful selection of HBDs and HBAs [17–19]. Hence, considering their low vapor pressure, excellent electrical conductivity and biocompatibility, DESs can be effectively used in electrochemical gas sensors and biosensors [16–22]. One of the most commonly used HBA is choline chloride (ChCl) in combination with different HBDs such as urea, oxalic, citric, succinic or amino acids, or glycerol (Gly) [16–19]. In this paper, a simple setup is proposed for the headspace electroanalytical measurement of volatile phenols in food matrices, such as wines, based on the combination of ChCl and Gly to form the DES used as non-aqueous supporting electrolyte. The direct electrochemical oxidation of phenolic compounds usually leads to polymeric product build-up on the electrode surface, so their previous oxidation to quinones catalysed by tyrosinase (TYR) enzyme is highlighted as alternative [17, 23]. The electrochemical reduction of the enzymatically produced quinones back to their original phenolic forms at cathodic potentials is associated with low noise and background currents, which leads to the detection of low analyte concentrations [23]. Thus, membrane-free TYR-based screen-printed carbon electrodes (SPCEs) have been developed for the detection of volatile phenols in wine, with both the enzyme and ChCl/Gly DES impregnated in paper crowns [13, 24]. The influence of modifying the working electrode with nanomaterials, such as AuNPs or C60, which have already been used for the detection of these phenolic compounds, has been studied prior to the enzyme immobilisation to record the maximum cathodic current.

## Materials and methods

### Reagents and materials

Analytical-reagent grade chemicals and Milli-Q water (Milli-pore, Bedford, MA, USA) were used. 0.1 M phosphate buffer solutions (KH_2_PO_4_, Fluka, Munich, Germany), adjusted to the appropriate pH by phosphoric acid (Panreac, Barcelona, Spain), or mixtures of ChCl and Gly from Sigma-Aldrich (Steinheim, Germany) were used as supporting electrolytes for electrochemical measurements.

One millimolar of HAuCl_4_ solutions was prepared by dissolving the appropriate amount of hydrogen tetrachloroaurate-(III) trihydrate (Acros Organics, Belgium) in 0.5 M sulfuric acid (Merck, Darmstadt, Germany).

C60 (Acros Organics, Geel, Belgium) solutions were prepared in dichloromethane (Panreac, Barcelona, Spain) and activated in 1.0 M potassium hydroxide solutions (Carlo Erba, Val de Reuil, France).

The filter paper used for supporting the DESs was purchased from Labor (Cordenons, Italy) as 50 cm × 50 cm foils, 67 g/m^2^. Circular crowns (Ø = 8 mm outside and Ø = 3 mm inside) were cut from these foils using hammer blades from KS Tools (Milan, Italy) [13].

TYR enzyme (8540 units/mg, Sigma-Aldrich, St. Louis, USA) was dissolved in 0.1 M phosphate buffer pH 6.5 [17].

Standard solutions of 4-ethylphenol (Alfa Aesar, Haverhill, Massachusetts, USA), 4-ethylguaiacol (Alfa Aesar, Haverhill, Massachusetts, USA), 4-vinylphenol (BLD Phamatech Ltd., Kaiserslautein, Denmark) and phenol (Sigma-Aldrich, Steinheim, Germany) were prepared by dissolving the appropriate amount of each reagent in phosphate buffer pH 6.5. A first dilution of 100 mg/L was prepared in phosphate buffer pH 6.5 and, then, a second one of 10 mg/L was prepared in DES for the voltammetric behaviour studies of each phenol.

Electrochemical measurements were carried out using an EmStat3 potentiostat (Palmsens BV, Houten, The Netherlands) and SPCEs based on a 3-electrode configuration, with 4-mm diameter carbon working, carbon counter and Ag/AgCl reference electrodes (DRPC11L, Metrohm DropSens, Oviedo, Spain).

### Preparation of DES

DES was synthesised using ChCl and Gly in a stoichiometric ratio of 1:2 as described in the literature [17]. The mixture was stirred and heated to 80 °C for 2 h.

### AuNPs modification of SPCEs

The electrochemical deposition of AuNPs onto the carbon working electrode was performed in 0.5 M H_2_SO_4_ containing 1 mM HAuCl_4_ by applying a potential of + 180 mV for 150 s, according to a previously reported method [25].

### C60 modification of SPCEs

C60-modified SPCEs were developed by drop-casting 40 μL of a 0.1 mg/mL solution of C60 in dichloromethane onto the carbon working electrode surface and allowed to dry at room temperature, according to previously reported methods. The original neutral C60 has been reported to behave as an insulator with insufficient porosity to allow the electroactive compounds to reach the working electrode surface. Thus, to enhance its low electrochemical activity, an activation step was carried out by cycling the potential between 0 and − 1.5 V at 10 mV/s in a 1 M KOH solution [7, 26, 27].

### Enzymatic sensor assembly

**Fig. 1:**
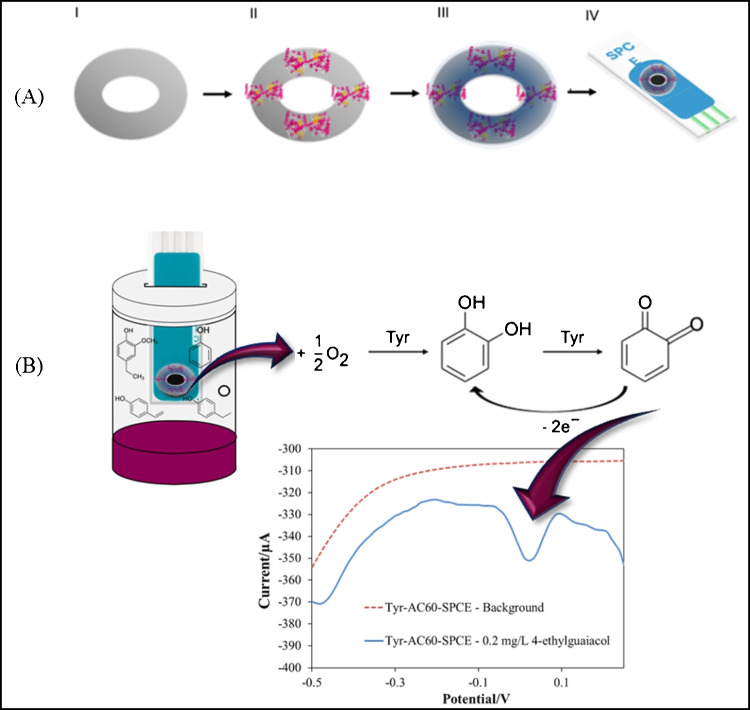
**A** Schematic representation of electrode modifications: (I) paper crown, (II) paper crown modification with TYR enzyme, (III) TYR/paper crown soaked in DES and (IV) DES/TYR/paper crown placed on a SPCE. **B** Schematic representation of the headspace electrochemical measurements carried out using a DES/TYR/paper crown placed on a SPCE

The measurement setup was based on SPCE, AuNPs/SPCEs or AC60/SPCEs suitably modified using a crown paper on which the enzyme was immobilised and soaked with DES, as schematically represented in Fig. 1A. In detail, 10 μL of an 8.5 units/μL solution of TYR was drop-casted on the paper crown and left to evaporate. After allowing the enzyme solution to dry, the paper was soaked in 20 μL of DES, eliminating excess DES by sorption onto the filter paper. Finally, the paper crown was placed onto the 3-electrode configuration device, connecting the outer edge of the carbon disk working electrode, as well as the peripheral counter and reference electrodes, giving rise to TYR/SPCE, TYR/AuNPs/SPCEs or TYR/AC60/SPCEs.

### Headspace electrochemical measurements

Enzymatic sensors were placed at the top of a sealed cell, manufactured using a UV resin for 3D printers with a side hole that allowed the introduction of samples [10], containing 1 mL of phosphate buffer pH 6.5. Contact between the enzymatic sensor and solution in the cell was avoided. After the addition of a volume of the phenol standard solution into the cell, TYR could oxidise the molecules at the headspace in equilibrium with the liquid phase, and subsequently, the reduction current of the produced orthoquinone could be recorded by voltammetry or chronoamperometry (Fig. 1B).

## Results and discussion

**Fig. 2:**
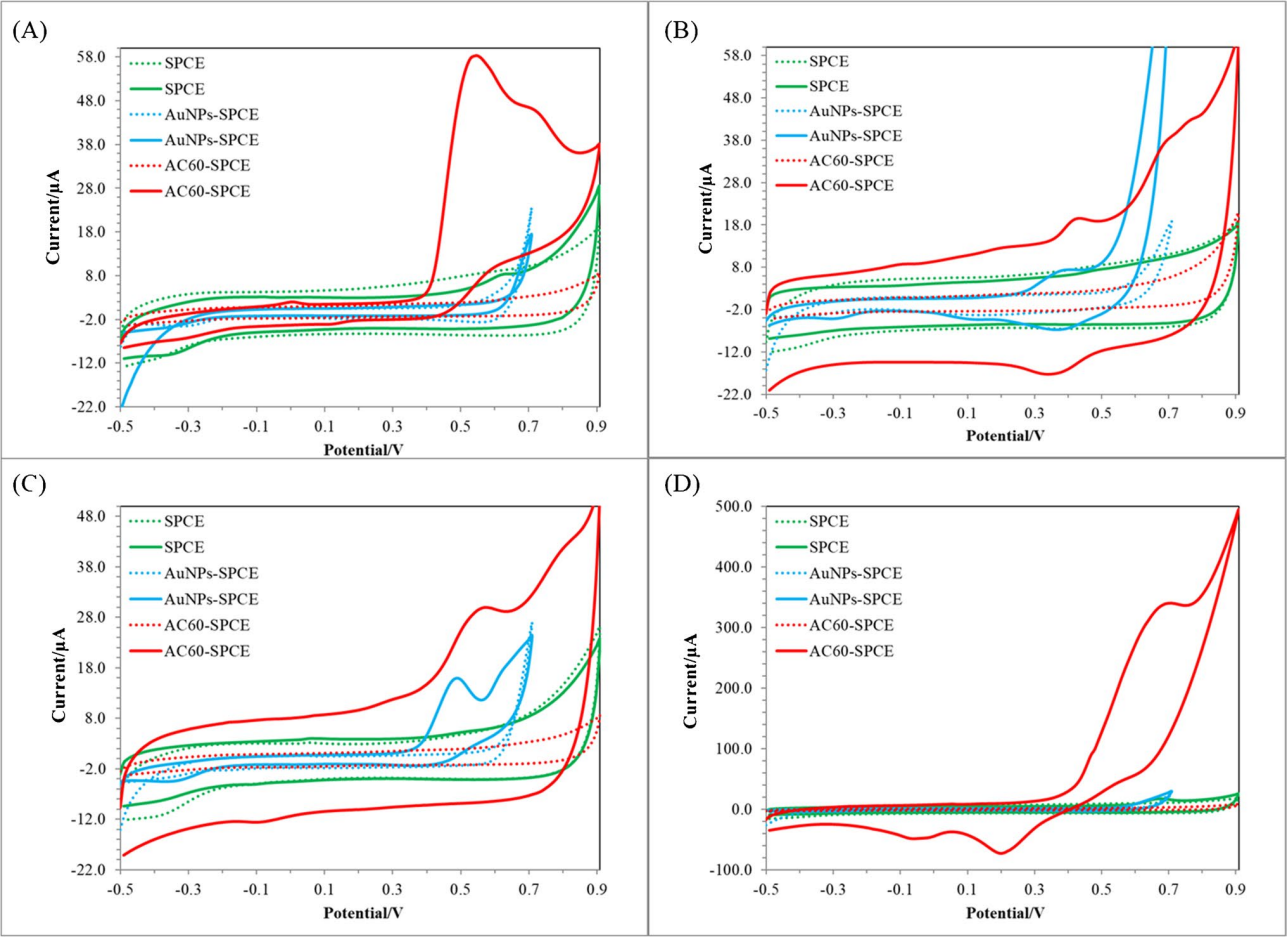
Cyclic voltammograms of 10 mg/L solutions of **A** 4-ethylphenol, **B** 4-ethylguaiacol, **C** phenol and **D** vinylphenol in DES at SPCE, AuNPs/SPCE and AC60/SPCE. Scan rate, 50 mV/s. The voltammograms in the dotted lines correspond to the blank solutions

First, voltammetric analyses were performed using different transducers to determine the electrochemical window of the enzymatic sensors that will be developed on them for the headspace detection of volatile phenols. To assess the behaviour of 4-ethylphenol (Fig. 2A), 4-ethylguaiacol (Fig. 2B), phenol (Fig. 2C) and 4-vinylphenol (Fig. 2D), cyclic voltammetric measurements were performed using unmodified SPCEs, AuNPs/SPCEs and AC60/SPCEs in solution. Measurements were performed by drop-casting 50 µL of 10 mg/L of the corresponding phenol in the DES solution at a scan rate of 50 mVs^−1^. In the case of AuNPs/SPCEs, the potential was not scanned further than + 0.7 V to prevent oxidation of AuNPs. The redox behaviour of the 4 phenols was enhanced by using the nanomaterial-modified SPCEs, registering anodic currents at higher potentials than + 0.25 V, with a considerable reduction in the capacitive current when using AuNPs/SPCEs. Consequently, AuNPs/SPCEs and AC60/SPCEs were considered suitable for the development of TYR-based sensors.

**Fig. 3:**
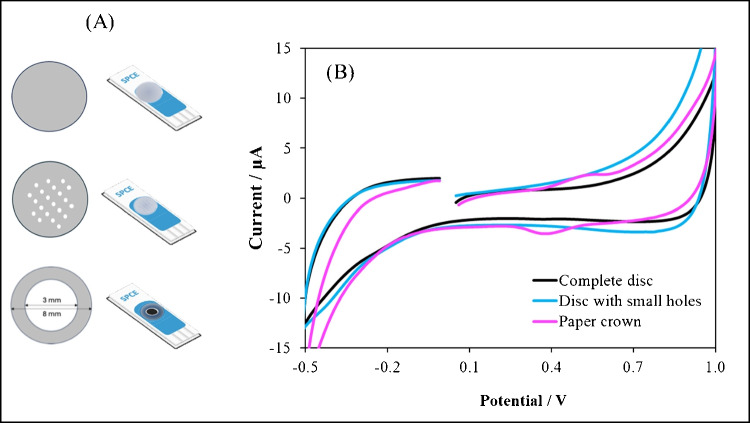
**A** Filter paper shapes tested for the placement of DES as supporting electrolyte for phenol electrochemical measurements using SPCEs. **B** Cyclic voltammograms recorded in the gas phase for a phenol standard of approx. 100 µg/L at SPCEs with DES-impregnated a complete disc, a disc with small holes and a paper crown. Scan rate, 50 mV/s

The key point for the headspace electroanalytical measurement of these volatile phenols is the placement of the supporting electrolyte in the devices. Initially, DES was deposited directly on the three electrodes of the SPCEs devices; however, no voltammetric signals due to phenols were observed. Therefore, the DES-impregnated filter paper was tested to provide contact between the three electrodes. The low density and high porosity of the filter paper may also facilitate subsequent enzyme immobilisation and oxygen permeation [13]. The paper shape, that is, complete discs, discs with small holes and paper crowns, was optimised to ensure that the recorded current was not affected by the blockage in the diffusion of the volatile analyte through the DES (Fig. 3A). The good redox behaviour of a 100 µg/L phenol solution was only observed when using the DES-impregnated paper crowns (Fig. 3B), so this configuration was considered optimum for the following detection of volatile phenols using headspace TYR-based sensors.

**Fig. 4:**
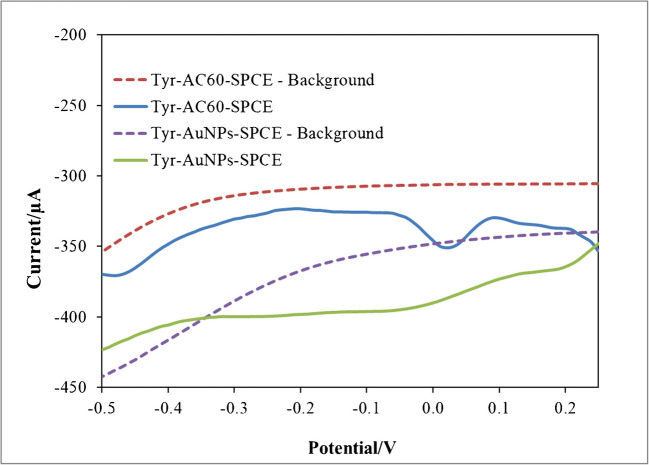
Headspace differential pulse voltammograms recorded for 0.2 mg/L 4-ethylguaiacol solutions at TYR/AuNPs/SPCEs and TYR/AC60/SPCEs with DES-impregnated paper crowns. Step potential, 0.01 V; pulse potential and time, 0.2 V and 0.02 s; scan rate, 50 mV/s

Membrane-free TYR-based SPCEs were suggested to detect these volatile compounds in wines, avoiding their direct electrochemical oxidation at the electrode surface, since polymeric product build-up could affect its sensibility [17, 23]. In this way, the analytical response would be based on the electrochemical reduction of the enzymatically produced orthoquinone (Fig. 1B). Since this cathodic current was perfectly registered for 4-ethylguaiacol solutions at AuNPs/SPCEs and AC60/SPCEs (Fig. 2B), this phenolic compound was taken as a representative of the volatile ones in wine for headspace voltammetric and chronoamperometric measurements. As mentioned above, the oxidation of 4-ethylguaiacol at AuNPs/SPCEs or AC60/SPCEs occurred at a potential higher than + 0.25 V (Fig. 2B), so any cathodic current recorded from this potential towards more negative potentials should be attributed entirely to the reduction of the enzymatically produced orthoquinone. Thus, differential pulse voltammograms of 4-ethylguaiacol solutions were recorded after an incubation period of 10 min using TYR/AuNPs/SPCEs and TYR/AC60/SPCEs from + 0.25 V to − 0.5 V. It could be seen in Fig. 4 two well-separated one-electron reduction processes, already described for quinones, at more positive potentials than − 0.1 V [28, 29]. Consequently, the entire reduction process would be easily registered at a constant potential of − 0.1 V by chronoamperometry. This potential was taken as the optimum value for recording the cathodic current related to the presence of 4-ethylguaiacol by chronoamperometry.

**Fig. 5:**
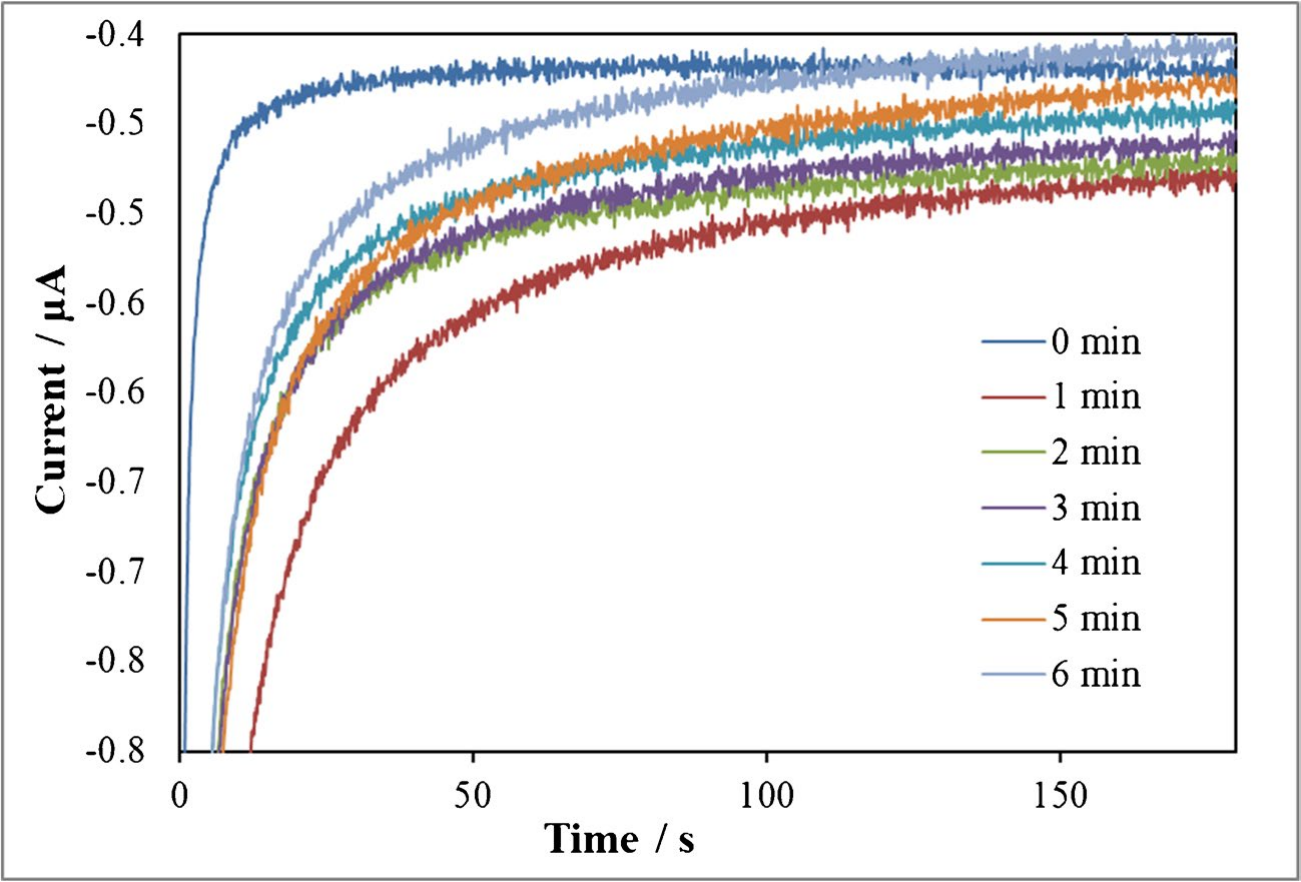
Headspace chronoamperograms recorded for a 200 µg/L 4-ethylguaiacol solutions at a TYR/AuNPs/SPCE with a DES-impregnated paper crown. Applied potential, − 0.1 V

**Fig. 6:**
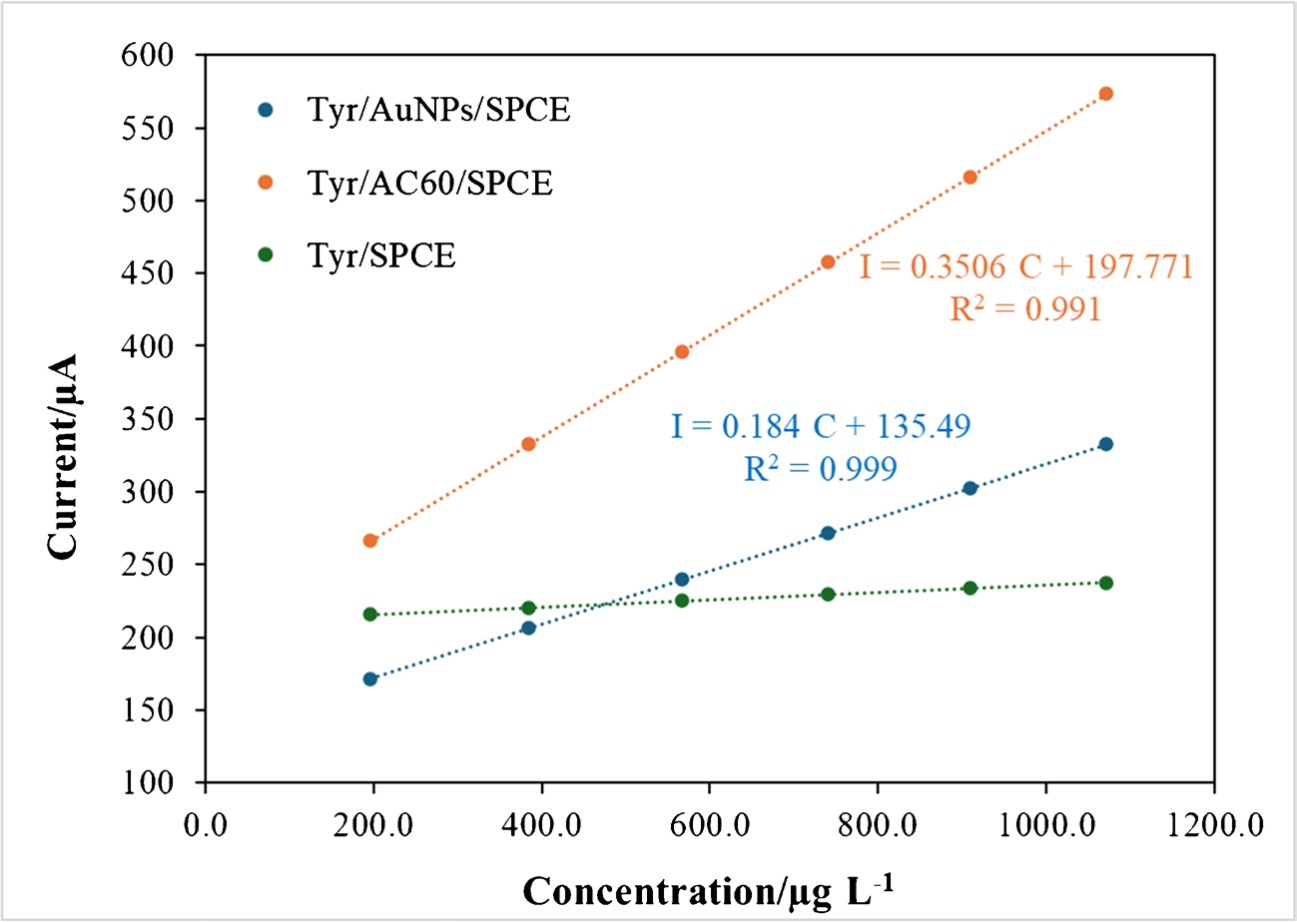
Experimental points obtained for different concentrations of 4-ethylguaiacol solutions, and the corresponding calibration lines, using TYR/SPCEs, TYR/AuNPs/SPCEs and TYR/AC60/SPCEs with DES-impregnated paper crowns

The incubation time, that is, the enzymatic reaction time for the production of the orthoquinone, was optimised as well in order not to enlarge the analysis time. The intensity of a 0.2 mg/L 4-ethylguaiacaol solution was recorded using a TYR/AuNPs/SPCE for 180 s at 1-min intervals. As shown in Fig. 5, the maximum cathodic current was registered after an incubation period of 1 min for the interaction between TYR and the volatile phenol. Then, headspace chronoamperograms were recorded for different concentrations of 4-ethylguaiacol solutions using TYR/SPCEs, TYR/AuNPs/SPCEs and TYR/AC60/SPCEs with DES-impregnated paper crown, after an incubation period of 1 min, at − 0.1 V. The highest sensitivity for the detection of volatile phenols was obtained by using the enzymatic sensors based on AC60 (Fig. 6). Thus, the variation in current at TYR/AC60/SPCE, reflecting the enzymatic activity of tyrosinase on the electrode surface, was analysed to quantify the volatile phenols in wine.

**Fig. 7:**
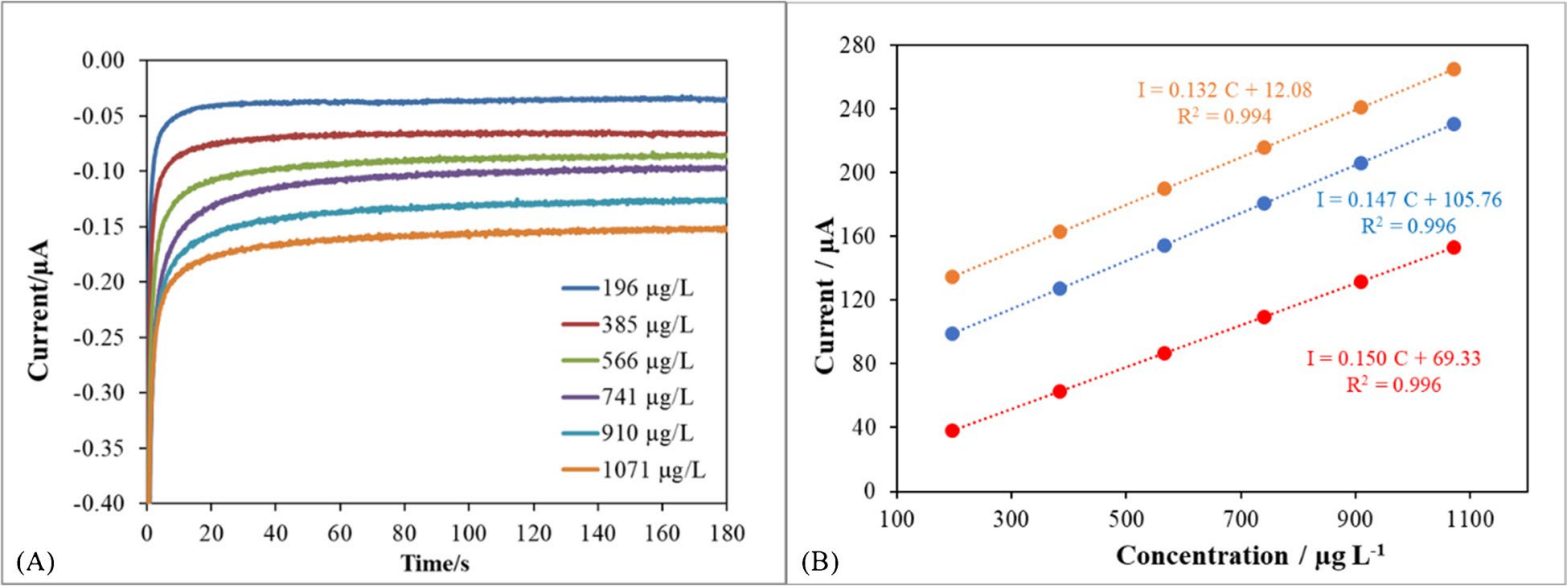
**A** Headspace chronoamperograms recorded for different 4-ethylguaiacol solutions at a TYR/AC60/SPCE with a DES-impregnated paper crown. Applied potential, − 0.1 V. **B** Experimental points obtained for different concentrations of 4-ethylguaiacol solutions, and the corresponding calibration lines, using three different TYR/AC60/SPCEs with DES-impregnated paper crown

The performance of these sensors was tested in terms of precision, by means of the relative standard deviation (RSD) of the slopes obtained from several calibration curves (intensity read at 120 s vs. concentration) (Fig. 7A). This approach ensures more accurate results by avoiding reliance on a single concentration, and allows the variability of measurements to be assessed [30]. Outlier points with a studentised residual above 2.5 in absolute value were removed to provide a proper evaluation of the calibration parameters obtained by ordinary least squares regression [31]. An RSD of 7.2% was statistically measured, expressing the relative variability of a three calibration curves recording in the 4-ethylguaiacol concentration range from 0.2 to 1.1 mg/L, indicating the consistency of the results (Fig. 7B).

In addition, the lowest concentrations of analyte that can be detected with certainty above the background noise was estimated. These measurements are crucial for assessing the sensitivity of the sensor and its ability to detect compounds in real samples such as wine. The limit of decision (CCα) and capability of detection (CCβ) were determined based on a linear regression model according to ISO 11843, using the DETARCHI program, instead of the signal-to-noise (S/N) ratio method for CCα [32–34]. The probability of false positive (α), that is, of detecting erroneously the analyte when it is not in the sample, was fixed at a value of 0.05, which led to a CCα of 92 µg/L (α = 0.05). CCβ was calculated considering not only the probability of false positive (α), but also the negative one (β), that is, the probability of incorrectly not detecting the analyte when it is present. Similarly, a CCβ value of 175 µg/L was set for α = β = 0.05.

Finally, the trueness of this method was tested by spiking and recovering different wine samples [30]. Four wine samples from different grape varieties were studied in their original state and after addition of a known mass of 4-ethylguaiacol to a test portion. Headspace chronoamperograms were registered for the wines using TYR/AC60/SPCEs with DES-impregnated paper crown at − 0.1 V after an incubation period of 1 min. The developed procedure was straightforwardly applied to these complex samples, avoiding the usual extraction pretreatments required for red wines when using AC60/SPCEs in solution [8]. No increase in current was recorded due to the oxidation process of 4-ethylguaiacol at the surface of the electrode; therefore, it was stated that there were no volatile phenols in the samples in their original state. Recovery experiments were then performed by analysing the fortified wine samples using the standard addition method. Headspace chronoamperograms were recorded for the corresponding wine and successive additions of a 4-ethylguaiacol solution to the cell. As shown in Table 1, the concentrations found in these samples agreed with that added, resulting in good recovery values. Although recoveries significantly different from unity, lower and/or higher, indicate that a bias affects the method, deviations lower than 20% are understood as sufficient to guarantee trueness [30, 35]. The percentage of relative standard deviation (RSD) of these measurements, performed in triplicate, calculated from their standard deviation and average, was lower than 12%. These results highlight the applicability of the developed analytical method for the detection of volatile phenols in wines.

**Table 1:** Recovery experiment results for the headspace determination of 4-ethylguaiacol in different wine samples using TYR/AC60/SPCEs with a DES-impregnated paper crow

Wine sample	Concentration added (µg/L)	Concentration found (µg/L)	Recovery (%)	RSD (%) (*n* = 3)
White wine (Macabeo and Gewürztraminer varieties)	177	199 ± 31	112	12
Red wine (Tempranillo and Syrah varieties)	177	187 ± 21	106	8
White wine (Chardonnay variety)	177	187 ± 24	105	9
Red wine (Merlot and Syrah varieties)	177	190 ± 25	107	10

## Conclusions

A novel tyrosinase-based gas sensor was developed for the detection of volatile phenolic compounds. The use of a DES electrolyte guarantees an appropriate environment for headspace electrochemical measurements, which is a simple and green strategy for the development of this type of enzymatic sensors. The use of a paper crown modified with the enzyme tyrosinase, soaked in DES and placed on SPCEs was found to be the best configuration for sensor design development. AuNPs and C60 nanomaterials clearly influence the redox behaviour of different phenolic compounds. TYR/AC60/SPCEs were the transducers that showed the highest sensitivity for the detection of 4-ethylguaiacol as a representative of volatile phenols, with a capability of detection below the perception threshold of this compound in wines. The reproducibility of the enzymatic sensor in the analysis of both synthetic and fortified wine samples was also adequate, showing successful recovery values between 105 and 112%, without the need for prior sample treatment. Thus, the obtained results demonstrate that this novel enzymatic sensor can successfully replace classical analytical methods and suggest a future application for the in situ monitoring of phenol content in wineries.

## Data Availability

Data is provided within the manuscript or supplementary information files.
